# Unraveling the power of sense of coherence: a key predictor of symptom severity among depressive disorders clients

**DOI:** 10.1186/s12912-024-02587-4

**Published:** 2025-01-07

**Authors:** Samah Mohamed Taha, Mona Metwally El-Sayed, Mahmoud Abdelwahab Khedr, Ayman Mohamed El-Ashry, Azza Ibrahim Abdelraof, Hassan Mohammed Sonbol, Manal Mohammed Hawash, Eman Sameh Abd Elhay

**Affiliations:** 1 Psychiatric and Mental Health Nursing, Faculty of Nursing, Mansoura University, Mansoura, Egypt; 2 Psychiatric and Mental Health Nursing, Faculty of Nursing, Alexandria University, Alexandria, Egypt; 3 Psychiatry, Faculty of Medicine, Mansoura University, Mansoura, Egypt; 4 Gerontological Nursing, Faculty of Nursing, Alexandria University, Alexandria, Egypt

**Keywords:** Sense of coherence, Predictor, Symptom severity, Depressive disorders

## Abstract

**Background:**

Despite advancements in therapies, the severity of depressive symptoms continues to challenge psychiatric nurses. However, research has indicated that a higher sense of coherence (SOC) can act as a protective factor against the development of diseases and promote faster recovery.

**Aim:**

This study aimed to investigate whether SOC could serve as an indicator of depressive symptom severity among clients with depression.

**Design:**

The study employed a cross-sectional design and followed the STROBE checklist for reporting observational studies.

**Methods:**

A convenient sample of 169 clients with depression participated in the study. The researchers utilized the Antonovsky Sense of Coherence Scale-29 and the Beck Depression Inventory-II to assess SOC and depressive symptoms, respectively.

**Results:**

The study findings indicated that a substantial proportion of the participants (35.5%) had low Sense of Coherence (SOC) levels, associated with higher depressive symptoms. The severity of depression showed a significant negative correlation with difficulties in understanding and finding meaning in life and challenges in effectively coping with life’s demands (*r* = -0.626, *p* < 0.001). The regression analysis revealed that for every one-unit increase in SOC, there was a corresponding decrease of 0.5 units in depressive symptoms. The SOC variable accounted for 39.1% of the variance in depressive symptoms severity.

**Conclusion:**

Psychiatric nurses should consider implementing interventions to enhance SOC, such as Life Skills Training (LST), psychoeducation, cognitive-behavioral therapy, and mindfulness practices, particularly for depressive disorder clients with a poor sense of coherence.

**Supplementary Information:**

The online version contains supplementary material available at 10.1186/s12912-024-02587-4.

## Introduction

Depression is a common mental condition, affecting 10% of the general population over their lifetime. It can occur up to 20% of the time in clinical settings [1]. Major depressive disorder (MDD) substantially impairs psychosocial functioning and lowers quality of life, with an elevated risk of death. According to the World Health Organization (WHO), major depression was ranked as the third leading cause of disease burden globally in 2008 [2]. It is projected that by 2030, major depression will become the leading cause of disease burden worldwide [3]. Depressive diseases typically begin in adolescence, peaking between the ages of 15 and 24. In the Diagnostic and Statistical Manual of Mental Disorders (DSM-5) published by the American Psychiatric Association, the severity of major depressive disorder (MDD) is categorized as mild, moderate, or severe [4]. This classification is determined by considering factors such as the number of symptoms present, the level of distress caused by the symptoms, and the extent of impairment in social and occupational functioning [5].

Determining the severity of an illness has important clinical implications. The severity of the disorder influences treatment decisions, including whether to seek therapy, the type and degree of treatment, and whether to continue or discontinue treatment [6]. Expectations for role function and disability status are also influenced by severity. In therapy trials, severity measures are utilized to assess treatment outcomes, and their relevance extends to clinical practice, where they can serve as valuable endpoints [7]. An estimated 30% of people with depressive diseases do not respond to several evidence-based therapies, and their depression is labeled “treatment resistant“ [8]. Many clients are at high risk of recurrence, with a 60% lifetime risk following the first severe depressive episode. Up to 70% of people with two major depressive episodes will have recurrences throughout their lives, and 90% of those with three or more will have more recurring episodes. Furthermore, one-third to half of the clients reported recurrence within one year of discontinuing treatment [9].

The depressive disorder’s unpleasant emotional experiences shorten attention spans, lessen the capability for flexible and creative thought, and lessen the capacity for adaptive behavior. This restricts one’s capacity to cope with stressful conditions now and in the future [10]. A generalized and largely stable dispositional orientation, or what is known as the Aaron Antonovsky sense of coherence (SOC), which is unique to a particular person and determines the fundamental way of perceiving, feeling, and understanding the world around one and one’s own life, can also affect the choice of adaptive coping mechanisms [11, 12].

Sense of coherence comprises comprehension, manageability, and meaningfulness [13, 14]. The degree to which people understand and make meaning of life experiences is called comprehensibility. An individual who understands what is going on can better deal with challenging conditions. The extent to which people believe they have enough available (internal and external) resources to meet their requirements is defined as manageability. The sense of meaningfulness reflects individuals’ perception of emotional value in their lives and their ability to view the difficulties they encounter as challenges rather than obstacles, thus motivating them [15]. Numerous studies have underscored the critical role of a sense of coherence in promoting health [12, 16–18].

A sense of coherence has a protective effect on psychological well-being and is related to resilience and hardiness [19]. It has been found that clients with psychiatric diagnoses of depressive, neurologic, psychotic, and anxiety disorders have lower levels of SOC than those who do not [20, 21]. According to research conducted by Izydorczyk (2019), a higher sense of coherence, precisely a sense of comprehensibility, was linked to a lower intensity of harmful and overall psychiatric symptoms [22].

Unquestionably, a person with significant major depression disorders may go through substantial stress levels brought on by a recurrence of the illness, the accumulation of psychopathological symptoms, and the requirement for additional hospitalization [23]. Research reported that a higher sense of coherence shields people against developing diseases and, should they do so, speeds up their recovery [24]. Moreover, cultural norms, values, and beliefs in Egypt play a significant role in shaping how individuals experience mental health and approach seeking help. The Egyptian culture is characterized by collectivism, strong familial bonds, and an emphasis on social relationships [25]. These cultural factors impact how individuals perceive their symptoms, their strategies to cope with mental health challenges, and their overall sense of well-being. Thus, the influence of SOC on symptom severity among individuals with depressive disorders within the specific cultural context of Egypt remains understudied.

Psychiatric nurses play a crucial role in managing depressive symptoms and enhancing the sense of coherence in individuals with depression [26]. They assess symptom severity, develop treatment plans, and use supportive communication to improve comprehensibility. Their medication management and psychotherapy roles contribute to treatment manageability and foster a sense of meaningfulness. They also provide evidence-based counseling services to help clients challenge negative thoughts and develop coping strategies [27]. Continuous monitoring, crisis intervention, and resilience promotion are integral to their role, helping clients manage symptoms and build necessary resources for recovery. By advocating for clients’ rights and needs, they foster a sense of coherence, aiding in alleviating depressive symptoms and improving overall well-being. The study aimed to determine if SOC levels are a reliable indicator of symptom intensity in Egyptian clients with major depressive disorders.

## Research hypothesis

Clients with higher levels of sense of coherence would experience lower levels of depressive symptom severity.

## Materials

### Study design and setting

A cross-sectional correlational research design was followed in the study over three months, from Jun 1 to the end of August 2023.

### Study setting

The psychiatric outpatient clinics at El-Mansoura University Hospital, affiliated with the Faculty of Medicine at El-Mansoura University, offer free treatment to individuals with mental illnesses. These clinics cover a range of specialized areas, including general psychiatry and substance use disorders. Services provided at the clinics encompass mental health evaluation, diagnosis, prescription of medications, and counseling. The study was conducted within the outpatient clinic. A comfortable room was selected for the research, ensuring privacy and confidentiality during client sessions. The clinic works all week except Friday from 8 a.m. to 1 p.m.

### Study participants

#### Sample size estimation

Based on hospital statistics for 2022–2023, the psychiatric outpatient clinics recorded a daily range of one to two clients with depressive disorders, totaling approximately 72 to 144 clients over three months. To determine the sample size for the study, the G*Power Windows 3.1.9.7 program was utilized, considering specific criteria. These criteria encompassed an effect size of 0.5, an alpha error probability of 0.05, a power of 0.90 (1 - error probability), an examination of a single group, and a single measurement. The study included a total of 169 participants who had been diagnosed with a depressive disorder based on the criteria specified in the DSM-5 [1, 4, 28].

#### Inclusion and exclusion criteria

The study set specific eligibility criteria to ensure the participants were suitable for the research. To be eligible for participation in the study, individuals were required to be outpatient clients who met the diagnostic criteria for depressive disorders as specified in the Fifth Edition of the Diagnostic and Statistical Manual for Mental Disorders (DSM-5) [4]. Additionally, the participants had to be at least 18 years old. Furthermore, the participants were expected to be able to communicate coherently and meaningfully.

Clients with certain comorbid conditions, such as dementia, schizophrenia, bipolar I or II disorder, anxiety disorders, or other psychotic disorders, were excluded as part of the study’s exclusion criteria. Additionally, clients with a history of substance use within the past two years were excluded. These exclusion criteria aimed to ensure that these comorbid conditions or substance use did not significantly influence the manifestation or assessment of depressive disorders.

### Recruitment process

**Fig. 1:**
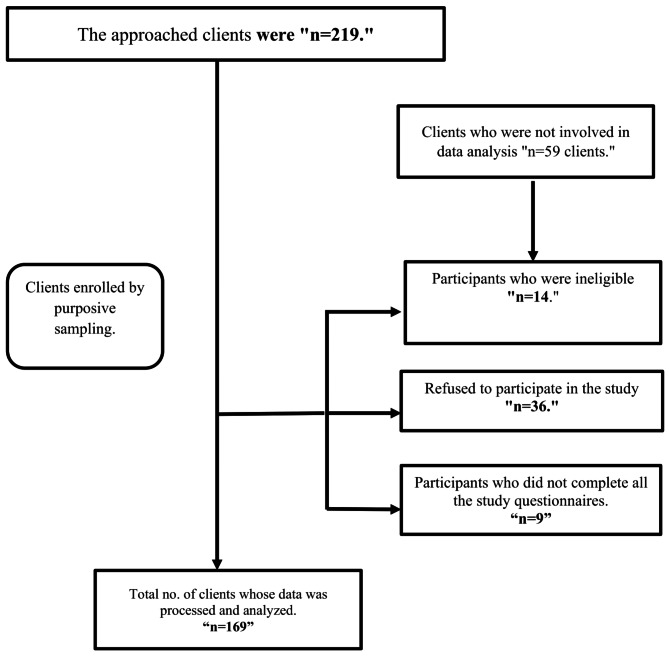
Participants’ recruitment flow chart

The participants for this study were recruited using a purposive sampling technique. The medical records were retrieved to determine their eligibility. Initially, 227 clients with depressive disorders were identified. However, after the screening process, it was found that 14 individuals were ineligible due to specific criteria, an additional 36 clients refused to participate, and 9 clients still needed to complete all the questionnaires. 169 clients who willingly agreed to participate in the study were enrolled as participants (Fig. 1).

### Study measures

#### Sociodemographic and clinical sheet

The researchers developed a questionnaire to collect sociodemographic and clinical data from the participants. This tool aimed to gather information regarding the client’s age, gender, employment status, marital status, and educational background. Furthermore, the questionnaire also included inquiries about physical complaints, the onset of depressive disorder, the number of hospitalizations, methods of treatment such as pharmacological interventions, psychotherapy, or electroconvulsive therapy (ECT) sessions, and the presence of depressive disorders in the family history.

#### Antonovsky sense of coherence scale (SOC-29)

The SOC-29 is a self-rated questionnaire developed by Antonovsky (1993) [14]. The scale assesses three components of a sense of coherence: comprehensibility, manageability, and meaningfulness. Participants were asked to rate each item on the SOC-29 scale using a 7-point Likert scale, where 1 represented “strongly disagree” and seven represented “strongly agree.” The scoring range of the SOC-29 scale extends from 29 to 203, with higher scores indicating a greater level of coherence. The scale provides Cutoff points to facilitate the interpretation of the overall score. A score between 51 and 100 suggests a low sense of coherence, a score between 101 and 152 indicates an average sense of coherence, and scores above 152 suggest a high sense of coherence. The SOC was deemed valid and produced a Cronbach’s alpha score of 0.75 [29]. Two bilingual researchers translated the adopted English version of the SOC-29 scale into Arabic. The Arabic version was then independently back-translated into English by two other researchers. To determine the factor structure and content validity of the translated scale, an Exploratory Factor Analysis (EFA) was performed. The goodness of fit indices, such as CFI (Comparative Fit Index), TLI (Tucker-Lewis Index), and RMSEA (Root Mean Square Error of Approximation), were employed to evaluate the adequacy of the model fit. The results showed a CFI of 0.872, TLI of 0.829, and RMSEA of 0.100. The scale’s internal consistency was evaluated using Cronbach Alpha, yielding a value of 0.88.

#### Arabic version of Beck depression inventory (BDI-II)

The BDI-II, developed by Beck et al. (1996), is a self-report assessment comprising 21 items [30]. It is designed to evaluate the level of depression severity in both adult and adolescent populations. The items of the BDI-II evaluate symptoms that align with the diagnostic criteria for depressive disorders as outlined in the Diagnostic and Statistical Manual of Mental Disorders. The items on the questionnaire are rated using a 4-point Likert scale, where participants indicate the severity of symptoms on a scale from 0 (no symptoms) to 3 (severe symptoms). The total score on the BDI-II can range from 0 to 63, with higher scores indicating a greater severity of depressive symptoms. The scoring categories are as follows: a total score of 0–13 is considered Mild mood disturbance, 14–19 is classified as borderline clinical depression, 20–28 as moderate, and 29–63 as severe. The standardized Arabic version was used in this study (Alansari, 2006) [31] and demonstrated excellent internal consistency, with a Cronbach’s alpha coefficient of 0.91.

### Procedure

#### Ethical approvals

The Research Ethical Committee (REC) of El-Mansoura University’s Faculty of Nursing granted ethical approval for the research, following the Code of Ethics of the World Medical Association as stated in the Declaration of Helsinki. The study obtained approval from the Hospital for Psychiatric Medicine manager and the Mental Health Secretariat to uphold ethical standards. A comprehensive review of the client’s medical charts was conducted to determine the participants’ eligibility.

Before their participation, all participants were presented with a transparent explanation of the study’s objectives and comprehensive details about the questionnaire. Informed written consent was obtained from each participant, ensuring their understanding and voluntary agreement to participate in the study. Participants were assured of the confidentiality of their information. They were explicitly informed of their right to decline participation or withdraw from the study at any time without facing any repercussions. No financial incentives were provided to the participants for their involvement in the study.

### Tool validity and reliability

Before the primary data collection, a pilot study was conducted to validate and establish the reliability of the data collection measures. The pilot study involved 10 clients diagnosed with depressive disorders who were excluded from the overall sample. Its purpose was to evaluate the clearness of the data collection instruments and identify any potential concerns or obstacles that could arise during the data collection.

The researchers conducted Cronbach’s Alpha coefficient test to assess the reliability of the SOC-29 and BDI-II scales. The test results indicated a respectable level of reliability for both scales, indicating their consistency in measuring the intended constructs.

The researchers translated the SOC-29 scale into Arabic to make it applicable to Arabic culture. The content validity of the translated SOC-29 scale was assessed through confirmatory factor analysis. A standardized Arabic version of the BDI-II scale was utilized in the study. Seven experts from the Department of Psychiatric Nursing within Alexandria and El Mansoura faculties were assembled to review and evaluate the research tools to ensure face validity and relevance.

### Data collection process

During the data collection process, one to two face-to-face structured interviews were conducted with each client to gather the required information for the study. The duration of each interview, typically lasting between 20 and 25 min, varied based on factors such as the client’s attentiveness, focus, and comprehension.

### Data processing and analysis

Data analysis was performed using Statistical Package for the Social Sciences (SPSS) version 26.0 after coding and computerizing the dataset. The data were subjected to a cleaning process and assessed for normality using the Shapiro test. Quantitative data were summarized using mean and standard deviation, while qualitative data were presented in terms of percentages and numerical counts. Significance was determined at a 5% level of significance. The reliability of the study instruments was assessed according to their internal consistency using Cronbach’s alpha test. Furthermore, the relationships between depressive symptoms and a sense of coherence were examined using the Pearson correlation coefficient. Additionally, the influence of a sense of coherence on depressive symptoms was analyzed using simple linear regression.

## Results

Table 1 shows that 68.0% of patients fall within the 20–30 age range, followed by 23.1% in the 30–40 range. Male patients constituted 59.8%, while 40.2% were females. The table also reveals that 60.4% of the studied patients were single and resided in urban areas. Most of the patients (60.9%) had a secondary level of education. The table reveals that 66.9% of patients had one child, and 64.5% had more than four family members. Most patients (85.8%) considered their income sufficient. Working patients constituted 54.4%, and those who complained of physical diseases represented 42.6% of the studied patients. The onset of depression for 61.5% of patients was from less than five years, and 68% were admitted to the hospital at least one time. Finally, the table indicates that most patients (81.7%) received medication as a form of treatment, 8.3% received psychotherapy, and 10.0% underwent ECT sessions. Most patients (82.8%) had no family history of depression.

**Table 1:** Sociodemographic characteristics of patients (No = 169)

Sociodemographic characteristics	No.	%
Age		
< 20	7	4.1
20–30	115	68.0
30–40	39	23.1
> 40	8	4.8
M (SD)	**27.60 (6.20)**	
Gender		
Male	101	59.8
Female	68	40.2
Marital status		
Single	80	47.3
Married Divorced	67 22	39.6 13.1
Residence		
Rural	67	39.6
Urban	102	60.4
Education		
Primary	39	23.1
Secondary	103	60.9
University	27	16.0
Children number		
One	113	66.9
2 or More	56	33.1
M (SD)	**0.99 (1.31)**	
Family number		
< 5	60	35.5
5 or More	109	64.5
M (SD)	**4.64 (1.17)**	
Monthly Income		
Sufficient	145	85.8
Insufficient	24	14.2
Living condition		
Alone	12	7.1
With family members	157	92.9
Occupation		
Working	92	54.4
Not working	77	45.6
Physical disease		
Yes	72	42.6
No	97	57.4
Onset years of depressive disorder		
< 5	104	61.5
5 or More	65	38.5
M (SD)	**3.63 (2.43)**	
Number of Hospitalizations		
One	115	68.0
2 or More	54	32.0
M (SD)	**0.97 (0.83)**	
Methods of Treatment		
Pharmacological	138	81.7
Psychotherapy	14	8.3
Electro-convulsive sessions	17	10.0
Family history		
Yes	29	17.2
No	140	82.8

Table 2 demonstrates that 49.7% of the studied patients reported a low level of comprehensibility, and 29.0% reported a high level, with a mean score of 39.69 (10.33). It also shows that 37.9% of the studied patients reported a moderate level of manageability, and 26.6% reported a high level, with a mean score of 33.86 (5.76). More than half (52.7%) of the studied participants reported a low level of meaningfulness, and only 14.2% reported a high level, with a mean score of 29.49 (5.63). Looking at the overall SOC score, the mean of 103.03 with a standard deviation of 6.50 suggests a moderate sense of coherence among the participants. Specifically, nearly two-thirds (64.5%) of the participants reported a moderate level of SOC, and no individuals reported a high level.

**Table 2:** Means, Standard Deviations, and levels of Sense of Coherence (No = 169)

	M	SD	Low		Moderate		High	
			No	%	No	%	No	%
Comprehensibility	39.69	10.33	84	49.7	36	21.3	49	29.0
Manageability	33.86	5.76	60	35.5	64	37.9	45	26.6
Meaningfulness	29.49	5.63	89	52.7	56	33.1	24	14.2
Total of SOC-29	103.03	6.50	60	35.5	109	64.5	0	0.0

SOC: Sense of Coherence

Low (51–100).   Moderate (101–152).   High (more than 152)

Table 3 illustrates that 36.1% of the studied patients had moderate depression, followed by 30.2% for those with borderline clinical depression. Patients who had mild mood disturbance constituted 23.7%, while those who had severe depression represented 10.1%.

**Table 3:** Means, standard deviations, and levels of Beck-Depression Inventory II (No = 169)

	Mild mood disturbance		Borderline clinical depression		Moderate depression		Severe depression	
	No	%	No	%	No	%	No	%
BDI-II	40	23.7	51	30.2	61	36.1	17	10.1
M (SD)	**21.36 (5.81)**							

BDI-II: Beck-Depression Inventory

Mild mood disturbance (0–13).   Borderline clinical depression (14–19).   Moderate (20–25), 39, 42, 43).   Severe (29–63)

Table 4 presents compelling evidence of a significant negative correlation between Comprehensibility, Manageability, and Meaningfulness with the Total Score of BDI-II. This suggests that individuals with higher levels of these factors are likely to experience fewer depressive symptoms (Comprehensibility: *r* = -0.447, *p* < 0.001; Manageability: *r*=-0.542, *p* < 0.001; Meaningfulness: *r* = -0.456, *p* < 0.001). Furthermore, the total score of SOC-29 is also negatively associated with the total score of BDI-II, indicating that individuals with a stronger sense of coherence are likelier to have lower depressive symptoms (*r* = -0.626, *p* < 0.001).

**Table 4:** Correlations Coefficient Matrix between the sense of coherence domains on SOC-29 and depressive symptoms severity on BDI-II (No = 169)

		Comprehensibility	Manageability	Meaningfulness	Total of SOC	Total of BDI-II
Comprehensibility	**r**					
	**P**					
Manageability	**r**	0.735^*^				
	**P**	> 0.001^*^				
Meaningfulness	**r**	0.204^*^	0.280^*^			
	**P**	0.008	> 0.001^*^			
Total of SOC-29	**r**	0.762^*^	0.525^*^	0.293^*^		
	**p**	> 0.001^*^	> 0.001^*^	> 0.001^*^		
Total of BDI-II	**r**	-0.447^*^	-0.542^*^	-0.456^*^	-0.626^*^	
	**p**	> 0.001^*^	> 0.001^*^	> 0.001^*^	> 0.001^*^	

SOC: Sense of Coherence.   BDI-II: Beck-Depression Inventory

r: Pearson coefficient   *: Statistically significant at *p* ≤ 0.05

Table 5 showcases the results of a robust linear regression analysis conducted to predict the severity of depressive symptoms. The model uses the total score on the Beck Depression Inventory (BDI-II) as the dependent variable and the sense of coherence as the independent variable. The results of the statistical analysis indicate a significant negative relationship between the Sense of Coherence and the severity of depressive symptoms. For every unit increase in SOC-29, the severity of depressive symptoms decreases by 0.559 units (B = -0.559, *p* < 0.001). This relationship is statistically significant and explains approximately 39.1% of the variance in depressive symptoms severity (R² = 0.391, F = 107.432, *p* < 0.001). The Durbin-Watson statistic of 2.133 suggests no autocorrelation in the residuals. The 95% confidence interval for the unstandardized coefficient B ranges from − 0.666 to -0.453, further confirming the significance of the relationship.

**Table 5:** Linear regression analysis between depressive symptoms severity (BDI-II) and sense of coherence (SOC-29) (No = 169)

	^a^. Depressive Symptoms Severity					
Variable	B	Beta	t	*p*	95% CI	
					LL	UL
^b^. Sense of Coherence (SOC-29)	-0.559	-0.626	-10.365^*^	< 0.001^*^	-0.666	-0.453
R^2^ = 0.391, F = 107.432^*^, *p* < 0.001^*^, DW = 2.133						

a.: dependent variable (Depressive Symptoms Severity), b: independent variable (sense of coherence)

F: ANOVA test R^2^: Coefficient of determination.   B: Unstandardized Coefficients

Beta: Standardized Coefficients.   t: t-test of significance LL: Lower limit UL: Upper Limit

DW: Durbin-Watson < 2.0 positive autocorrelation

Durbin-Watson = 2.0 zero autocorrelation

Durbin-Watson > 2.0 negative autocorrelation

*: Statistically significant at *p* ≤ 0.05

## Discussion

The findings of the study revealed that a significant percentage of the participants (35.5%) had low levels of SOC, which were linked with heightened depressive symptoms. Additionally, the study showed that the severity of depression was negatively associated with difficulties in understanding and finding meaning in life, as well as challenges in effectively coping with life’s demands. The regression analysis uncovered that an increase of one unit in SOC corresponded with a decrease of 0.5 units in the severity of depressive symptoms. The SOC variable was responsible for 39.1% of the variation in the severity of depressive symptoms.

The present study found that a substantial portion of the examined clients reported low comprehensibility. Comprehensibility refers to how individuals perceive the world as structured, predictable, and understandable. Low comprehension can lead to confusion and uncertainty, exacerbating depressive symptoms [32]. Similar findings are observed for manageability, where 37.9% of the patients reported a moderate level, indicating difficulties in managing life’s demands. Individuals with lower manageability may struggle with coping strategies, potentially leading to heightened depression symptoms [33]. The results also highlighted a particularly concerning aspect of meaningfulness, with 52.7% of participants reporting a low level. Meaningfulness refers to the degree to which life is perceived as purposeful and significant. A lack of meaningfulness is associated with despair and hopelessness, which are core features of depression [33]. The mean SOC score of 103.03, SD = 6.50 suggests a moderate sense of coherence among the participants. Notably, every individual reported a low level of SOC. This finding underscores the need for interventions to enhance SOC in this population, as a strong SOC has been linked to better mental health outcomes [32].

The present study’s findings provide valuable insights into the distribution of depression severity among the studied patients. Understanding the prevalence of different levels of depression is crucial for tailoring interventions and providing appropriate care. The most significant percentage of patients (36.1%) falling into the category of moderate depression suggests that a substantial portion of the sample is experiencing significant depressive symptoms. Moderate depression can considerably impact an individual’s daily functioning and overall quality of life [5]. Healthcare professionals must recognize and address moderate depression promptly to prevent it from progressing to more severe forms. The prevalence of borderline clinical depression among 30.2% of patients indicates that a substantial portion of the sample is experiencing symptoms that are close to the clinical threshold for a formal diagnosis of depression. Individuals in this category may benefit from early intervention and support to prevent their condition from worsening [5]. Nearly a quarter of the studied patients have reported mild mood disturbances. While these individuals may not meet the criteria for a clinical diagnosis of depression, their symptoms can still impact their well-being and daily lives. It is essential to provide support and monitor individuals with mild mood disturbances to prevent the development of more severe depression [34]. However, the smallest percentage, 10.1% of patients experiencing severe depression, represents a significant concern. A high level of distress and an elevated risk of suicide are commonly observed in individuals experiencing severe depression [35].

The BDI score’s moderate negative correlations with comprehensibility and meaningfulness suggest that increases in depression severity are linked with difficulties in understanding and finding life meaningful. Additionally, the strong negative correlation between BDI score and manageability indicates that more severe depression is linked to decreased abilities to manage life’s challenges effectively [36]. Furthermore, SOC, in light of Antonovsky (1987) [11] & Yamazaki (2008) [37], refers to an individual’s perception that stressors in their life are predictable and challenges rather than overwhelming burdens, and they feel equipped with adequate resources to cope with them. Our study suggests that a strong SOC leads to a decrease in the severity of depressive symptoms.

The study’s linear regression analysis revealed a statistically significant and inverse relationship between the SOC and the severity of depressive symptoms. Patients with higher levels of SOC exhibited lower levels of depressive symptoms. This finding aligns with previous research consistently supporting the idea that a strong sense of coherence is a protective factor against the occurrence and severity of depressive symptoms [20, 21]. A higher sense of coherence is associated with improved psychological well-being and resilience, which can serve as a buffer against depression [24, 38]. Furthermore, a study conducted by Li et al. (2021) in China following the COVID-19 pandemic found that exposure to negative epidemic-related information, rather than positive information, was significantly associated with depression, anxiety, and stress. SOC was also correlated with depression, anxiety, and stress. A higher sense of coherence was protective in college students’ adjustment during the post-epidemic period [39].

The study yielded that SOC could account for 39.1% of the variability in depressive symptom severity. These findings suggest that SOC plays a significant role in understanding and predicting the severity of depressive symptoms in the studied patients. These results align with a previous study conducted by Moksnes et al. (2014), which also observed a strong and statistically significant negative association between SOC and depression in both genders. This association remained significant even after controlling for age and stress [24]. Further, Yano et al. (2019) [40] conducted a study in Japan. They indicated that a robust sense of coherence played a moderating role in the connection between sensory-processing sensitivity and depressive symptoms among university students. Moreover, a recent study by Li et al. (2023) conducted in China found that SOC partially mediated the relationship between perceived stress and depressive symptoms. The mediation analysis indicated that SOC accounted for 49.78% of the mediating effect, with a direct effect size of -0.23 (BCa95% CI: [-0.31, -0.14]). These findings support the importance of SOC in understanding the link between stress and depressive symptoms [22].

### Limitations

The study’s potential limitation lies in its relatively small sample size, which may restrict the generalizability of the findings to larger populations of individuals experiencing depressive symptoms. The study’s cross-sectional design involves collecting data at a specific moment, which restricts the ability to establish causality or track longitudinal changes in both the sense of coherence (SOC) and depressive symptoms. The study relies on self-reported measures, such as the Beck Depression Inventory (BDI), which can be subject to response bias, social desirability bias, or inaccuracies in self-assessment. Although the English version of SOC-29 was used in the study, it is recommended that the Arabic version of SOC-13 be used instead since it has already been tested for reliability and validity among Arabic participants. Other predicting variables that could influence SOC and depressive symptoms, such as past trauma, social support, or access to mental health services, may not have been included in the analysis.

## Conclusion

The study’s findings provide significant insights into the correlation between a sense of coherence (SOC), depressive symptoms, and the distribution of depression severity among Egyptian clients. The three components of SOC—comprehensibility, manageability, and meaningfulness—are interconnected and play crucial roles in an individual’s ability to cope with life stressors. A significant portion of the patients in the study exhibited moderate levels of comprehensibility, manageability, and meaningfulness, which could contribute to increased severity of depressive symptoms. BDI scores demonstrated moderately negative correlations with comprehensibility and meaningfulness, implying that higher depression severity is associated with difficulties in understanding and finding life meaningful. Severe depression was strongly correlated with decreased manageability, indicating challenges in effectively managing life’s demands. The linear regression analysis revealed a significant and inverse relationship between SOC and the severity of depressive symptoms. A higher SOC was correlated with low severity.

### Implications for psychiatric nursing

Assessment and Screening: Psychiatric nurses should routinely screen patients for depressive symptoms, considering the wide range of severity levels observed in this study. Early detection is critical for timely intervention. Individualized Care Plans: Tailored care plans should be developed based on an individual’s SOC profile, addressing specific areas of deficiency in comprehensibility, manageability, or meaningfulness. Interventions to Enhance SOC: Psychiatric nurses can play a pivotal role in implementing interventions to enhance SOC, such as psychoeducation, cognitive-behavioral therapy, and mindfulness practices. Strengthening an individual’s SOC can potentially mitigate depressive symptoms. Collaborative Care: Collaboration with other healthcare professionals, including psychologists and social workers, can ensure comprehensive and multidisciplinary support for patients experiencing depressive symptoms. Prevention and Education: Education programs and strategies targeting individuals at risk of depression, such as those with poor SOC, should be developed and implemented.

## Electronic supplementary material

Below is the link to the electronic supplementary material.

Supplementary Material 1

## Data Availability

The datasets used and analyzed during the current study are available from the corresponding author upon reasonable request.

## Abbreviations

BDI-II: Beck Depressive Inventory-II
ECT: Electro-Convulsive Therapy
LST: Life Skills Training
MDD: Major depressive disorder
SOC: Sense of Coherence
WHO: World Health Organization
