# Supplementary material for: Unraveling the power of sense of coherence: a key predictor of symptom severity among depressive disorders clients
Source: BMC Nurs. 2025 Jan 7;24:21. doi: 10.1186/s12912-024-02587-4 (PMC11707934; doi:10.1186/s12912-024-02587-4)
Supplement: Supplementary file 1 — Supplementary Material 1 [file 12912_2024_2587_MOESM1_ESM.docx]

**Sociodemographic and Clinical Sheet**

| 1. **Sociodemographic characteristics:** | |
| --- | --- |
| - **Age :----------------------------** |  |
| **- Gender:** |  |
| - Male | **( )** |
| - Female | **( )** |
| **- Marital Status:** |  |
| - Single | **( )** |
| - Married | **( )** |
| - Divorced | **( )** |
| - **Residence** |  |
| - Rural | **( )** |
| - Urban | **( )** |
| - **Education** |  |
| - Primary | **( )** |
| - Secondary | **( )** |
| - University | **( )** |
| - **Children number------------** |  |
| - **Family number---------------** |  |
| - **Income** |  |
| - Sufficient | **( )** |
| - Insufficient | **( )** |
| - **Living condition** |  |
| - Alone | **( )** |
| - With family members | **( )** |
| - **Occupation** |  |
| - Working | **( )** |
| - Not working | **( )** |
| 1. **Clinical data** |  |
| - **Physical disease** |  |
| - Yes | **( )** |
| - No | **( )** |
| - **Age at Onset of Depressive Symptoms:---------------** | |
| - **Hospital Admission number----------** |  |
| - **Past Treatment History:** |  |
| - Pharmacological | **( )** |
| - Psychological | **( )** |
| - Electro-convulsive sessions | **( )** |
| - **Family history** |  |
| - Yes | **( )** |
| - No | **( )** |

**The 29-item Sense of Coherence Scale**

|  | When you talk to people‚ do you have the feeling they don’t understand you? | 1  Never have this feeling. | 2 | 3 | 4 | 5 | 6 | 7  Always have this feeling. |
| --- | --- | --- | --- | --- | --- | --- | --- | --- |
|  | In the past‚ when you had to do something which depended upon cooperation with others‚ did you have the feeling that it: | 1  Surely wouldn’t get done. | 2 | 3 | 4 | 5 | 6 | 7  Surely would get done. |
|  | Think of the people with whom you come into contact daily‚ aside from the ones to whom you feel closest. How well do you know most of them? | 1  You feel that they’re strangers. | 2 | 3 | 4 | 5 | 6 | 7  You know them very well. |
|  | Do you have the feeling that you don’t really care what goes on around you? | 1  Very seldom or never | 2 | 3 | 4 | 5 | 6 | 7  Very often. |
|  | Has it happened in the past that you were surprised by the behavior of people whom you thought you knew well? | 1  Never happened. | 2 | 3 | 4 | 5 | 6 | 7  Always happened. |
|  | Has it happened that people whom you counted on disappointed you? | 1  Never happened. | 2 | 3 | 4 | 5 | 6 | 7  Always happened. |
|  | Life is | 1  Full of interest | 2 | 3 | 4 | 5 | 6 | 7  Just routine. |
|  | Until now your life has had | 1  No clear goals or purpose at all. | 2 | 3 | 4 | 5 | 6 | 7  Very clear goals and purpose. |
|  | Do you have the feeling that you’re being treated unfairly? | 1  Very often. | 2 | 3 | 4 | 5 | 6 | 7  Very seldom or never. |
|  | In the past 10 years your life has been: | 1  Full of changes without knowing what will happen next. | 2 | 3 | 4 | 5 | 6 | 7  Completely consistent and clear. |
|  | Most of the things you do in the future will probably be | 1  Completely fascinating. | 2 | 3 | 4 | 5 | 6 | 7  Deadly boring. |
|  | Do you have the feeling that you’re in an unfamiliar situation and don’t know what to do? | 1  Very often. | 2 | 3 | 4 | 5 | 6 | 7  Very seldom or never. |
|  | What best describes how you see life? | 1  One can always find a solution to painful things in life. | 2 | 3 | 4 | 5 | 6 | 7  There is no solution to painful things in life. |
|  | When you think about your life‚ you very often: | 1  Feel good to be alive. | 2 | 3 | 4 | 5 | 6 | 7  Ask yourself why you exist at all |
|  | When you face a difficult problem‚ the choice of a solution is: | 1  Always confusing and hard to find. | 2 | 3 | 4 | 5 | 6 | 7  Always completely clear |
|  | Doing the things, you do every day is: | 1  A source of deep pleasure and satisfaction. | 2 | 3 | 4 | 5 | 6 | 7  A source of pain and boredom. |
|  | Your life in the future will probably be: | 1  Full of changes without knowing what. | 2 | 3 | 4 | 5 | 6 | 7  Will happen next completely consistent and clear |
|  | When something unpleasant happened in the past your tendency was: | 1  “To eat yourself up” about it. | 2 | 3 | 4 | 5 | 6 | 7  To say “ok‚ that’s that‚ I have to live with it” and go on. |
|  | Do you have very mixed-up feelings and ideas? | 1  Very seldom or never. | 2 | 3 | 4 | 5 | 6 | 7  Very often. |
|  | When you do something that gives you a good feeling: | 1  It’s certain that you’ll go on feeling good. | 2 | 3 | 4 | 5 | 6 | 7  It’s certain that something will spoil the feeling. |
|  | Does it happen that you have feelings inside you would rather not feel? | 1  Very often. | 2 | 3 | 4 | 5 | 6 | 7  Very seldom or never. |
|  | You anticipate that your personal life in the future will be: | 1  Totally without meaning or purpose | 2 | 3 | 4 | 5 | 6 | 7  Full meaning and purpose. |
|  | Do you think that there will always be people whom you can count on in the future? | 1  You’re certain there’ll be. | 2 | 3 | 4 | 5 | 6 | 7  You doubt there’ll be. |
|  | Does it happen that you have the feeling that you don’t know exactly what’s about to happen? | 1  Very often. | 2 | 3 | 4 | 5 | 6 | 7  Very seldom or never. |
|  | Many people—even those with a strong ch‎aracter—sometimes feel like sad sacks (losers) in certain situations. How often have you felt this way in the past? | 1  Never | 2 | 3 | 4 | 5 | 6 | 7  Very often. |
|  | When something happened‚ you have generally found that: | 1  You overestimated or underestimated its importance. | 2 | 3 | 4 | 5 | 6 | 7  You saw things in the right proportion. |
|  | When you think of difficulties you are likely to face in important aspects of your life‚ do you have the feeling that: | 1  You will always succeed in overcoming the difficulties. | 2 | 3 | 4 | 5 | 6 | 7  You won’t succeed in overcoming the difficulties. |
|  | How often do you have the feeling that there’s little meaning in the things you do in your daily life? | 1  Very often | 2 | 3 | 4 | 5 | 6 | 7  Very seldom or never. |
|  | How often do you have feelings that you’re not sure you can keep under control? | 1  Very often | 2 | 3 | 4 | 5 | 6 | 7  Very seldom or never. |

**Beck's Depression Inventory**

1.

0 I do not feel sad.

1 I feel sad.

2 I am sad all the time and I can't snap out of it.

3 I am so sad and unhappy that I can't stand it.

2.

0 I am not particularly discouraged about the future.

1 I feel discouraged about the future.

2 I feel I have nothing to look forward to.

3 I feel the future is hopeless and that things cannot improve.

3.

0 I do not feel like a failure.

1 I feel I have failed more than the average person.

2 As I look back on my life, all I can see is a lot of failures.

3 I feel I am a complete failure as a person.

4.

0 I get as much satisfaction out of things as I used to.

1 I don't enjoy things the way I used to.

2 I don't get real satisfaction out of anything anymore.

3 I am dissatisfied or bored with everything.

5.

0 I don't feel particularly guilty.

1 I feel guilty a good part of the time.

2 I feel quite guilty most of the time.

3 I feel guilty all of the time.

6.

0 I don't feel I am being punished.

1 I feel I may be punished.

2 I expect to be punished.

3 I feel I am being punished.

7.

0 I don't feel disappointed in myself.

1 I am disappointed in myself.

2 I am disgusted with myself.

3 I hate myself.

8.

0 I don't feel I am any worse than anybody else.

1 I am critical of myself for my weaknesses or mistakes.

2 I blame myself all the time for my faults.

3 I blame myself for everything bad that happens.

9.

0 I don't have any thoughts of killing myself.

1 I have thoughts of killing myself, but I would not carry them out.

2 I would like to kill myself.

3 I would kill myself if I had the chance.

10.

0 I don't cry any more than usual.

1 I cry more now than I used to.

2 I cry all the time now.

3 I used to be able to cry, but now I can't cry even though I want to.

11.

0 I am no more irritated by things than I ever was.

1 I am slightly more irritated now than usual.

2 I am quite annoyed or irritated a good deal of the time.

3 I feel irritated all the time.

12.

0 I have not lost interest in other people.

1 I am less interested in other people than I used to be.

2 I have lost most of my interest in other people.

3 I have lost all of my interest in other people.

13.

0 I make decisions about as well as I ever could.

1 I put off making decisions more than I used to.

2 I have greater difficulty in making decisions more than I used to.

3 I can't make decisions at all anymore.

14.

0 I don't feel that I look any worse than I used to.

1 I am worried that I am looking old or unattractive.

2 I feel there are permanent changes in my appearance that make me look unattractive.

3 I believe that I look ugly.

15.

0 I can work about as well as before.

1 It takes an extra effort to get started at doing something.

2 I have to push myself very hard to do anything.

3 I can't do any work at all.

16.

0 I can sleep as well as usual.

1 I don't sleep as well as I used to.

2 I wake up 1-2 hours earlier than usual and find it hard to get back to sleep.

3 I wake up several hours earlier than I used to and cannot get back to sleep.

17.

0 I don't get more tired than usual.

1 I get tired more easily than I used to.

2 I get tired from doing almost anything.

3 I am too tired to do anything.

18.

0 My appetite is no worse than usual.

1 My appetite is not as good as it used to be.

2 My appetite is much worse now.

3 I have no appetite at all anymore.

19.

0 I haven't lost much weight, if any, lately.

1 I have lost more than five pounds.

2 I have lost more than ten pounds.

3 I have lost more than fifteen pounds.

20.

0 I am no more worried about my health than usual.

1 I am worried about physical problems like aches, pains, upset stomach, or constipation.

2 I am very worried about physical problems and it's hard to think of much else.

3 I am so worried about my physical problems that I cannot think of anything else.

21.

0 I have not noticed any recent change in my interest in sex.

1 I am less interested in sex than I used to be.

2 I have almost no interest in sex.

3 I have lost interest in sex completely.

INTERPRETING THE BECK DEPRESSION INVENTORY

Now that you have completed the questionnaire, add up the score for each of the twenty-one questions by counting the number to the right of each question you marked. The highest possible total for the whole test would be sixty-three. This would mean you circled number three on all twenty-one questions. Since the lowest possible score for each question is zero, the lowest possible score for the test would be zero. This would mean you circles zero on each question. You can evaluate your depression according to the Table below.

**Total Score_______________Levels of Depression**

1-10____________________These ups and downs are considered normal

11-16___________________ Mild mood disturbance

17-20___________________Borderline clinical depression

21-30___________________Moderate depression

31-40___________________Severe depression

over 40__________________Extreme
